# Redefining bioactive small molecules from microbial metabolites as revolutionary anticancer agents

**DOI:** 10.1038/s41417-023-00715-x

**Published:** 2024-01-10

**Authors:** Eileena F. Giurini, Aishvarya Godla, Kajal H. Gupta

**Affiliations:** 1 Division of Surgical Oncology, Department of Surgery, Rush University Medical Center, Chicago, IL 60612 USA; 2 Division of Pediatric Surgery, Department of Surgery, Rush University Medical Center, Chicago, IL 60612 USA

**Keywords:** Drug delivery, Cancer

## Abstract

Cancer treatment remains a significant challenge due to issues such as acquired resistance to conventional therapies and the occurrence of adverse treatment-related toxicities. In recent years, researchers have turned their attention to the microbial world in search of novel and effective drugs to combat this devastating disease. Microbial derived secondary metabolites have proven to be a valuable source of biologically active compounds, which exhibit diverse functions and have demonstrated potential as treatments for various human diseases. The exploration of these compounds has provided valuable insights into their mechanisms of action against cancer cells. In-depth studies have been conducted on clinically established microbial metabolites, unraveling their anticancer properties, and shedding light on their therapeutic potential. This review aims to comprehensively examine the anticancer mechanisms of these established microbial metabolites. Additionally, it highlights the emerging therapies derived from these metabolites, offering a glimpse into the immense potential they hold for anticancer drug discovery. Furthermore, this review delves into approved treatments and major drug candidates currently undergoing clinical trials, focusing on specific molecular targets. It also addresses the challenges and issues encountered in the field of anticancer drug research and development. It also presents a comprehensive exposition of the contemporary panorama concerning microbial metabolites serving as a reservoir for anticancer agents, thereby illuminating their auspicious prospects and the prospect of forthcoming strides in the domain of cancer therapeutics.

## Introduction

The use of microorganisms to find new drugs for human diseases has been a recurring practice for the past century. Following the discovery of penicillin from *Penicillium* mold in the 20^th^ century, thousands of bacteria and fungi strains were screened for new antibacterial antibiotics. The impact of these discoveries on humankind has been revolutionary, life expectancy was increased by nearly 30 years in the past century alone [1]. These breakthroughs can be attributed to two factors of microbial world: the sheer abundance of different microbial species, as well as these microorganisms providing a rich source of bioactive compounds that are produced by these organisms. The identification of approximately 22,500 bioactive compounds derived from microbes have led to the development of modern therapeutics beyond antibiotics [2]. Further exploration of these promising therapeutic compounds led to the discovery of novel set of bioactive molecules, which possess potential pharmacological properties.

Microbial metabolites comprise two main categories: primary and secondary. Primary metabolites are synthesized continuously, as they are vital for survival and important cellular processes including growth, development, and proliferation [3, 4]. For each of these processes primary metabolites can serve as signaling molecules, catalysts, stimulators, or inhibitors [2]. In contrast, secondary metabolites are not essential for survival or cellular function, rather are produced to confer an advantage to the organism in its environment. Secondary metabolites can serve as a chemical defense against environmental stressors or improve resource uptake [3–5]. Though typically dispensable to the organism that produces these molecules, many microbial derived-secondary metabolites have been discovered to have vast therapeutic application in humans [2]. Numerous secondary microbial metabolites have emerged as a significant reservoir for the production of antibiotics, antivirals, analgesics, and notably innovative anticancer therapies. Many synthetically developed cancer therapies are inspired by the secondary metabolites produced by microorganisms. In this review we will highlight the source and possible function of these compounds, the mechanism of action in mitigating cancer progression, as well as any limitations or challenges in the usage microbially derived metabolites as a treatment of cancer.

## Clinically established anticancer microbial metabolites

**Fig. 1:**
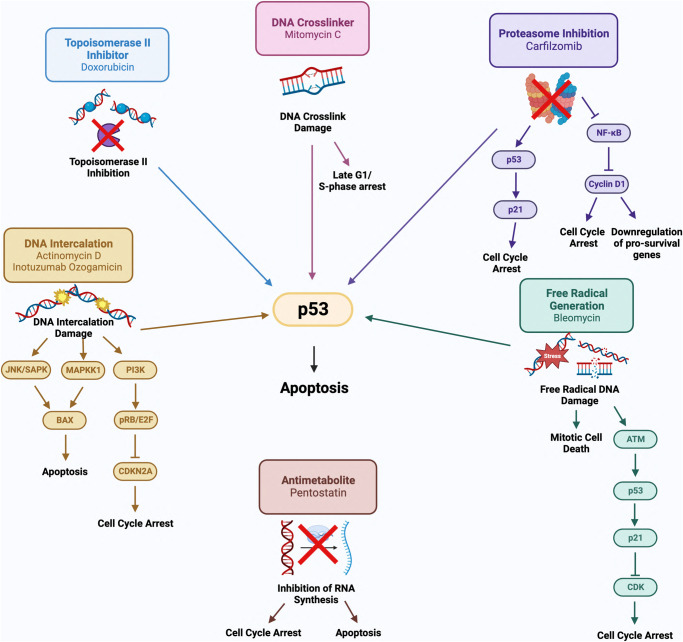
Molecular mechanisms of action for clinically established anticancer compounds derived from microbial metabolites. Topoisomerase II inhibitor doxorubicin activates p53 to trigger apoptosis in cancer cells. Mitomycin C induces DNA crosslink damage arresting the cell cycle in late G1/S-phase and activates p53 to induce apoptosis. Proteasome inhibitor carfilzomib activates and stabilizes p53 to arrest the cell cycle and induce apoptosis. Carfilzomib also inhibits the NF-κB pathway, leading to cyclin D1 inhibition, resulting in cell cycle arrest and downregulation of pro-survival genes. Bleomycin induces free DNA damage to induce cell cycle arrest, mitotic cell death, and activation of p53-mediated apoptosis. Pentostatin inhibits RNA synthesis inducing apoptosis and cell cycle arrest. Actinomycin D and the calicheamicin component of inotuzumab ozogamicin intercalate in DNA to induce apoptosis through p53, JNK/SAPK, and MAPKK1 signaling and induce cell cycle arrest through inhibition of CDKN2A. Created with Biorender.com.

By 1990, biologically active natural products comprised a significant portion of available pharmaceuticals [6]. This included several microbial metabolites discovered to have antineoplastic effects [7]. These compounds, known for their strong cytotoxic capabilities, have become an integral part of treatment for various types of cancers in modern medicine. In this section we comprehensively describe the mechanism of action and any associated toxicities of DNA intercalators (actinomycin D, inotuzumab ozogamicin), topoisomerase II inhibitors (doxorubicin), DNA crosslinkers (mitomycin C), proteasome inhibitors (carfilzomib), nucleoside analogs (pentostatin), DNA free radical inducers (bleomycins). The anticancer signaling cascades of these compounds are further described in Fig. 1.

### Actinomycin D

Actinomycin D, or dactinomycin is an antineoplastic antibiotic derived from *Streptomyces parvulus* [8]. The compound has demonstrated some bacteriostatic effect on various Gram-positive bacteria, however, its toxicity precludes its usage for antimicrobial applications. Consequently, actinomycin D has been indicated as an anticancer drug for Wilms’ tumor, rhabdomyosarcoma, Ewing’s sarcoma, testicular cancer, and ovarian cancer [8].

#### Mechanism of action

Actinomycin D exerts its cytotoxicity through DNA intercalation with its phenoxazone ring system at GpC sites and sustaining a slow rate of dissociation between actinomycin D and DNA. The DNA intercalation localizes the pentapeptide chains to the DNA minor groove, further stabilizing the actinomycin D-DNA interaction through hydrogen bonding. The actinomycin D-DNA interaction occurs when DNA is in a conformational intermediate state that is associated with the RNA transcriptional complex. Specifically, actinomycin D binds to guanine residues accessible from the transcriptional complex. Due to this binding RNA polymerase is blocked from further reading of template DNA, preventing elongation of RNA strands [9, 10]. Cancer cells are especially susceptible to actinomycin D due to high levels of transcription to accommodate rapid cell division. Though a highly effective anticancer compound, the usage of actinomycin D is limited due to its severe adverse effects. Adverse effects can include gastrointestinal ulceration, myelosuppression, anemia, soft tissue damage, and dermatological toxicities [8].

### Bleomycins

Bleomycins are glycopeptide antibiotics isolated from *Streptomyces verticullis* [5]. Bleomycins are structurally unique antineoplastic antibiotics; the compounds possess four discrete binding domains: a metal-binding domain, a DNA-binding domain, 4-amino-3-hydroxy-2-methylpentanoic acid connectivity domain, and a carbohydrate domain. Each domain contributes functionally to the cytotoxic activity of bleomycins. At the clinical level a combination of bleomycin A_2_ and B_2_ is used in the treatment of ovarian cancer, testicular cancer, Hodgkin’s and non-Hodgkin’s lymphoma, and squamous cell carcinomas of the head and neck [11].

#### Mechansim of action

Intravenous administration of bleomycins allow binding with Cu^+^ found in the blood serum, forming a bleomycin-Cu(II) complex. The bleomycin-Cu(II) complex is actively transported into cells, though the exact transporter has yet to be defined. Upon entry into the nucleus, bleomycin exchanges Cu(II) for Fe(II) to form a bleomycin-Fe(II) complex. The bleomycin-Fe(II) complex binds to O_2_, then accepts an electron to form the activated bleomycin complex- bleomycin-Fe(III)-OOH. This complex targets DNA through abstraction of the C4’ hydrogen atom from the deoxyribose of a pyrimidine nucleotide, forming a free radical intermediate at C4’. The subsequent steps leading to DNA breakage are determined by the presence or absence of O_2_. With O_2_ present, the C4’ radical reacts with O_2_ to form 4’-hydroperoxide through reduction of a peroxyl radical. 4’-hydroperoxide induces a series of chemical reactions culminating in DNA strand scission to form 3’-phosphoglycolate and 5’-phosphate termini. The O_2_-independent pathway leads to oxidation of the C4’ radical, forming a 4’-carbocation, then a C4’-oxidized abasic site through a hydroxylation reaction. Accordingly, single and double-stranded breaks in DNA are formed, leading to prevention of DNA synthesis [12, 13]. Activated bleomycins also have demonstrated a degree of sequence selectivity. Bleomycin-induced breakage was found to preferentially occur at 5’-GTGT*AC or 5’-TGT*A, where * is the cleavage site [14]. Unfortunately, sequence selectivity is not sufficient to mitigate the adverse effects of bleomycins. Pulmonary toxicity is one of the greatest limitations in the use of bleomycins, occurring in approximately 10% of patients treated with the drug. Bleomycin-induced lung injury can manifest in the form of pneumonitis or eosinophilic inflammation, which can progress to life-threatening pulmonary fibrosis [11].

### Carfilzomib

Carfilzomib is a proteasome inhibitor analog of epoxomicin, which is derived from *Actinomyces* strain No. Q996-17 [15]. This compound is a tetrapeptide epoxyketone, indicated for relapsed or refractory multiple myeloma [15]. Thus, carfilzomib shares many of the same structures as epoxomicin, including a peptide-binding portion and an epoxyketone pharmacophore [15]. It is these two structures that contribute to the anticancer activity of carfilzomib.

#### Mechanism of action

The peptide-binding portion allows covalent binding to the substrate binding regions of the 20 S proteasome, and the epoxyketone pharmacophore stereospecifically targets a catalytic threonine residue. This residue is located on the β5 chymotrypsin-like subunit of the proteasome, resulting in its irreversible inhibition. The proteasome inhibition of carfilzomib can drive several distinct cellular mechanisms that converge to the same result- cell death [16]. Proteasome inhibition also drives the accumulation and stabilization of proteins that are canonically rapidly degraded in cancer cells. Stabilization and activation of proapoptotic effectors Bak and Bax, allow for the formation of the apoptosome followed by activation of caspases and apoptosis pathways [17]. Similarly, carfilzomib-mediated proteasome inhibition also leads to stable expression of tumor suppressor p53 and its targets p21 and p27. As an inhibitor of cyclins CDK1, CDK2, and CDK4/6, enhanced intracellular levels of p21 prevents cell cycle progression from G1 to S-phase. Elevated p27 levels promote cell cycle arrest in G1 phase, followed by cell death [16, 17]. Clinical usage of carfilzomib is commonly associated with hypertension, dyspnea, peripheral neuropathy, and cardiac failure. Additionally, as identified in the FOCUS trial in which carfilzomib was administered with corticosteroids and cyclophosphamide, the carfilzomib arm experienced higher renal adverse events compared to the control arm (24% vs 9%, respectively) [16].

### Doxorubicin

Doxorubicin, an anthracycline antibiotic derived from actinobacterium *Streptomyces peucetius*, is amongst the most potent anticancer compounds [18]. Doxorubicin has a broad reach, the drug is used in the treatment of breast, lung, gastric, ovarian, thyroid, Hodgkin’s and non-Hodgkin’s lymphoma, multiple myeloma, sarcoma, and pediatric cancers [18].

#### Mechanism of action

In the cytoplasm, doxorubicin forms a doxorubicin-proteasome complex via binding to the proteasome’s 20 S subunit, allowing its entry into the nucleus through nuclear pore complexes [19]. Upon entry to the nucleus, doxorubicin can intercalate into DNA following a double-stranded break produced by Topoisomerase II, in effect stabilizing a Topoisomerase II-DNA complex and consequently preventing the double-stranded break from re-ligation [19]. Anthracycline antibiotics, including doxorubicin, possess a quinone moiety that can undergo redox reactions by oxidoreductases, producing the free radical semiquinone. Semiquinone can convert oxygen into reactive oxygen species (ROS). In cells, production of ROS can lead to lipid peroxidation and DNA damage, both of which induce apoptosis [19]. Another proposed mechanism of action pertains to doxorubicin-mediated chromatin damage. Upon intercalation in the DNA minor groove, the amino sugar groups of doxorubicin compete for space with histone H4 residues [20]. In effect, histones are “evicted” from nucleosomes, the foundation of chromatin. The resultant chromatin damage manifests as epigenetic and transcriptomic alternations as well as diminished DNA damage response [19, 20].

The double-edged sword of doxorubicin treatment while quite effective, its use is hindered by significant, dose-limiting toxicity. Cardiomyocytes, the cells responsible for contraction of the heart, are particularly susceptible to doxorubicin-mediated toxicity. Cardiomyocyte toxicity has been demonstrated by two mechanisms: apoptosis through upregulation of death receptors TNFR1, Fas, DR4, and DR5 and triggering ferroptosis through lipid peroxidation in mitochondria [21, 22]. At the clinical level, doxorubicin is associated with acute cardiotoxicity, which can develop into cardiomyopathy or congestive heart failure. In addition to cardiomyocytes, rapidly dividing cells are a target of doxorubicin. This can result in toxicity to red and white blood cells, gastrointestinal mucosa, and hair follicles resulting in myelosuppression, neutropenic enterocolitis, and hair loss, respectively [19].

### Inotuzumab ozogamicin

Inotuzumab ozogamicin is an antibody-drug conjugate containing an enediyne antibiotic component produced by *Micromonospora echinospora* [23]. It is an antineoplastic agent that comprises a humanized anti-CD22 monoclonal IgG4 antibody G544 that is conjugated to a cytotoxic antibiotic N-acetyl-gamma-calicheamicin through an acetyl butyrate linker. The antibiotic component of inotuzumab ozoagmicin belongs the calicheamicin class of antibiotics [23].

#### Mechanism of action

Following the binding of CD22, a surface antigen commonly expressed on B-cell malignancies, the ozogamicin component is internalized into the cell, forming an endosome. The encapsulated inotuzumab ozogamicin complex fuses with an intracellular lysosome. Due to the acidic environment within the lysosome, the inotuzumab ozogamicin complex is cleaved at the acetyl butyrate linker. As a result, the ozogamicin antibiotic is free from the anti-CD22 antibody, resulting in its activated form. Activated ozogamicin is released from the lysosome and migrates to the nucleus. Intranuclear ozogamicin intercalates into the minor groove of DNA. The DNA binding results in a structural change to the enediyne moiety of ozogamicin, leading to the formation of diradicals. These diradicals abstract hydrogen ions from the phosphodiester bonds of DNA, generating double stranded breaks through oxidative strand scission. Consequently, the cell cycle is arrested in G2/M-phase and triggers apoptotic cell death [23, 24]. Though the anti-CD22 monoclonal antibody component of inotuzumab ozogamicin confers a high degree of specificity in targeting B-cell acute lymphoblastic leukemia, liver toxicity has been linked to the drug. In the phase 3 INO-VATE study, hepatic veno-occlusive disease was observed in 11-13% in patients treated with inotuzumab ozogamacin compared to < 1% occurrence in the standard-treatment group [25].

### Mitomycin C

Mitomycin C (MMC) is a metabolite produced by *Streptomyces caespitosis* [26]. In bacteria, MMC is a highly potent antibiotic, inducing bacterial cell death through selective crosslinking of complementary strands of DNA [26]. Interestingly, the antibiotic and anticancer mechanisms of MMC are quite similar.

#### Mechanism of action

MMC undergoes a reductive activating reaction resulting in two alkylating reactions that occur in the cytosol of the cell. Bioactivation of MMC has been reported to be mediated by NRF2 in KEAP1-KO cancer cells [27]. The first activating reaction covalently links the C1 of MMC to DNA, and the second links DNA to MMC at C10, culminating in crosslinking of DNA. MMC typically forms crosslinks at the N6 position of adenine residues or the N2 or N7 position of guanine residues. The interaction between DNA and MMC prevents access to DNA synthesis machinery, inducing cell cycle arrest and cell death [26, 28]. Higher concentrations of MMC have also been shown to suppress RNA synthesis and protein translation, likely potentiating the cytotoxic properties of MMC [29]. A unique feature of MMC is that it can discriminate between cancerous and non-cancerous tissue. The selectivity of MMC relies on the hypoxic environment found within the core of many tumors. In such an oxygen poor environment, MMC can feasibly undergo activation via reduction and exert its cytotoxic properties [26]. In noncancerous tissue, where oxygen is significantly more abundant, MMC remains in its inactive form [26]. Consequently, noncancerous tissue experience much less of the cytotoxic effects of MMC. The selective cytotoxicity of MMC has made it a suitable treatment for bladder, breast, anal, and gastrointestinal malignancies [30].

### Pentostatin

Pentostatin, or 2’-deoxycoformycin, is an antimetabolite derived from *Streptomyces antibioticus*. Structurally, pentostatin mimics the nucleoside adenosine, though differs in that the purine ring is modified with a 7-membered ring in place of a 6-membered ring, as well as a deletion of the amino group from the purine ring that is replaced with a hydroxyl group.

#### Mechanism of action

Pentostatin enters cells through nucleoside transporters hENT1 and hENT2 [31]. Pentostatin potently binds and inhibits the enzyme adenosine adeaminase (ADA). ADA converts adenosine and deoxyadenosine and into inosine and deoxyinosine, respectively. Though expressed in all tissues, lymphoid cells have especially high ADA activity, thus ADA inhibitor pentostatin is indicated for the treatment of hairy cell leukemia. Mechanistically, ADA inhibition leads to increased intracellular levels of adenosine and deoxyadenosine, as ADA is unable to catabolize these nucleosides. To reduce the intracellular deoxyadenosine concentration, deoxyadenosine is converted to deoxyadenosine triphosphate (dATP). The increased intracellular levels of dATP negatively regulate ribonucleotide reductase activity, which results in decreased production of other nucleotides. Due to the lack of available deoxyribonucleotides, both DNA and RNA synthesis is obstructed, ultimately leading to cell death [32]. Consequently, pentostatin is regarded as a cell cycle selective agent, inducing cell cycle arrest in the DNA and RNA synthesis phase, S-phase [32, 33]. Pentostatin has also demonstrated synergistic capabilities when combined with cyclophosphamide and rituximab, culminating in enhanced therapeutic response in chronic lymphocytic leukemia with minor treatment-related toxicity [34].

## Emerging anticancer agents from microbial metabolites

**Fig. 2:**
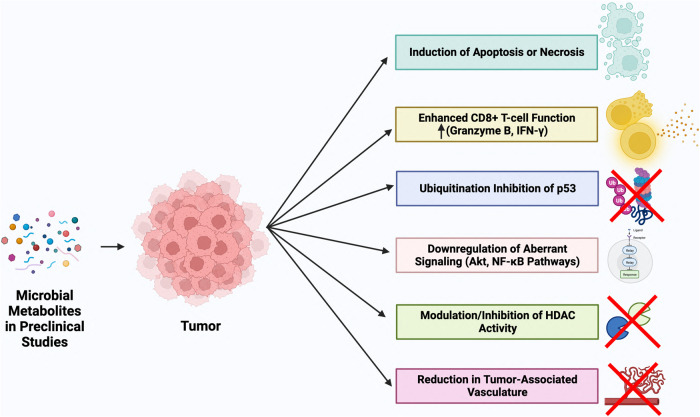
Mechanisms of action of anticancer compounds derived from microbial metabolites being evaluated in preclinical studies. The diverse range of metabolites produces also provide a number of discrete cancer-fighting mechanisms. These compounds can trigger programmed-cell death pathways such as apoptosis or necrosis (azurin-p28, butyrate, cordycepin, nisin), stimulate tumor-specific cytotoxic T-cells (butyrate, inosine, propionate), or maintain intracellular p53 levels by preventing p53 ubiquitination (azurin-p28). In addition, these compounds can target signaling pathways frequently dysregulated in cancer such as the PI3k-Akt and NF-κB pathways (cordycepin), inhibition of histone deacetylases (HDACs) (butyrate), and inhibition of tumor-associated angiogenesis (nisin). Created with Biorender.com.

Given the immense quantity and diversity of bioactive molecules produced by microorganisms, microbial metabolites provide a seemingly endless supply of compounds to be screened for anticancer potential. Table 1 exhibits the vast diversity in structure, class, and anticancer mechanism encompassed by these promising microbial metabolites. This section also highlights several microbial metabolites that have demonstrated promising antitumor activity in preclinical studies and early-stage clinical trials. The mechanisms of action of these compounds are also graphically displayed in Fig. 2. Anticancer compounds that have recently completed or ongoing clinical trials are further described in Table 2.

**Table 1:** List of secondary microbial metabolites studied in vitro and at the preclinical level and their experimental usage and mechanism of action.

Chemical or protein structure	Name	Class	Microbial source	Experimental studies	Target or mechanism of action	Reference
	3-hydroxydecanoic acid	Polyhydroxyalkanoate	*Pseudomonas putida*	MiaPaCa cells (pancreatic cancer), HeLa cells (cervical cancer), A549 cells (lung adenocarcinoma), WM793 cells (melanoma)	Induction of apoptosis	[60, 61]
	5-(*sec*-butyl)−2-ethylfuran-3-carboxylic acid	Furan-type Compound	*Streptomyces* sp. VN1	AGS cells (gastric adenocarcinoma), U87MG cells (glioblastoma), A549 cells (lung adenocarcinoma), A375SM cells (melanoma), HCT-116 cells (colorectal cancer)	Induction of cell death and inhibition of cell invasion activity	[62]
	Alborixin	Polyether Ionophore Antibiotic	*Streptomyces scabrisporus*	HCT-116 cells (colorectal cancer)	Induction of apoptosis via loss of mitochondrial membrane potential and ROS generation	[63]
	Asukamycin	Polyketide Antibiotic	*Streptomyces nodosus* subsp. *asukaensis*	231MFP cells (breast cancer)	Induction of apoptosis via functioning as molecular glue between p53 and UBR7	[64]
	Azalomycin F4A	Macrocyclic Lactone Antibiotic	*Streptomyces solisilvae*	MGC803 cells and xenograft (gastric cancer), AGS cells (gastric cancer)	Inhibition of autophagy via inhibition of ATG4B activity	[65]
	Bafilomycin A1	Macrolide Antibiotic	*Streptomyces griseus*	697 cells (pediatric B-ALL), patient-derived pediatric B-ALL cells	Induction of apoptosis via targeting apoptosis-inducing factor (AIF) pathway and inhibition of autophagy	[66]
	Bovicin HC5	Bacteriocin	*Streptococcus bovis*	MCF7 cells (breast cancer), HepG2 cells (hepatocellular carcinoma)	Induction of cell death	[67]
	Chromomycin A5	Aureolic Acid Antibiotic	*Streptomyces* sp. BRA-384	B16-F10 cells (melanoma)	Induction of apoptosis and immunogenic cell death via induction of autophagy and ER stress. Immunogenic cell death activates antigen presenting cells, T-cells, and release of DAMPs	[68]
	Chrysomycin A	Glycoside Antibiotic	*Streptomyces* sp. 891	U251 cells (glioblastoma), U87-MG cells (glioblastoma), NCI-H358 cells (NSCLC), Lewis lung carcinoma tumor model	Downregulation of Akt/GSK-3 β/β-catenin pathway to inhibit cell proliferation. Cell cycle modulator (S-phase arrest), induction of apoptosis via inhibition of Topoisomerase II enzyme	[69, 70]
Colicin A Colicin E1 Colicin U (Rendering NA)	Colicin A, E1, U	Bacteriocin	*Escherichia coli*	MCF7 cells (breast cancer), MDA-MB-231 cells (breast cancer), HS913T cells (fibrosarcoma), HOS cells (osteosarcoma)	Cell cycle modulator (G1 phase arrest), induction of apoptosis	[71]
	Colicin E7	Bacteriocin	*Escherichia coli*	HT-29 cells (colorectal cancer)	Upregulation of p53 and downregulation of bcl-2	[72]
	E492	Microcin	*Klebsiella pneumoniae*	HeLa cells (cervical cancer), Jurkat cells (immortalized T lymphocytes), RJ2.2.5 cells (Burkitt’s lymphoma)	Increasing intracellular calcium, induction of apoptosis and necrosis	[73, 74]
Glionitrin A Glionitrin B	Glionitrin A, B	Diketopiperazine Antibiotic	Antibiotic *Sphingomonas* strain KMK-001/*Aspergillus fumigatus* KMC-901 co-culture	HCT-116 cells (colorectal cancer), A549 cells (lung adenocarcinoma), AGS cells (gastric carcinoma), DU145 cells (prostate cancer)	Induction of cell death. Inhibition of cancer cell invasion and downregulation of MMP-2 and MMP-9	[75, 76]
	Kosinostatin	Quinocycline Antibiotic	*Streptomyces violaceusniger*	MCF-7 cells (breast cancer)	Induction of cell death, upregulation of p53	[77]
	L-Asparaginase	Enzyme	*Erwinia chrysanthemi, Escherichia coli*	MiaPaCa-2 (pancreatic cancer), PANC-1 (pancreatic cancer), patient-derived T- and B- ALL xenograft model	Glutamine metabolism via glutaminase activity, induction of cell death	[78, 79]
	Landomycin E	Angucycline Antibiotic	*Strepotomyces globisporus*	A2780 cells (ovarian cancer), HeLa cells (cervical cancer)	Induction of apoptosis via intracellular glutathione depletion	[80]
	Laterosporulin10	Bacteriocin	*Brevibacillus* sp. SKDU10	HeLa cells (cervical cancer), MCF-7 cells (breast cancer)	Induction of apoptosis via cell membrane permeabilization	[81]
	Lobophorin F	Spirotetronate Antibiotic	*Streptomyces* sp. SCSIO 01127	SF-268 cells (glioma), MCF-7 cells (breast cancer), NCI-H460 cells (lung large cell carcinoma)	Induction of cell death	[82]
	Manumycin A	Polyketide Antibiotic	*Streptomyces parvulus*	231MFP cells (breast cancer), SW480 cells and xenograft (colorectal cancer), Caco-2 cells (colorectal cancer)	Induction of apoptosis via functioning as molecular glue between p53 and UBR7 Induction of apoptosis via ROS generation and inhibition of PI3K-Akt signaling	[64, 83]
	Mensacarcin	Polyketide	*Streptomyces bottropensis*	SK-Mel-28 cells (melanoma), SK-Mel-5 cells (melanoma), HCT-116 cells (colorectal cancer)	Induction of caspase-dependent apoptosis via impairment of mitochondrial function Inhibition of glucose uptake and glycolysis impairment	[84, 85]
	Monensin	Polyether Antibiotic	*Streptomyces cinnamonensis*,	Panc-1 cells and xenograft (pancreatic cancer), MiaPaCa cells (pancreatic cancer), VCaP cells (prostate cancer), LNCaP cells (prostate cancer)	Cell cycle modulator (G1 phase arrest), induction of apoptosis through downregulation of EGFR. ROS accumulation to initiate apoptosis	[86, 87]
	Neoantimycin F	Depsipeptide	*Streptomyces conglobatus*	PC9 cells (lung adenocarcinoma), H1299 cells (NSCLC)	Cell cycle modulator (G1 or S-phase arrest), induction of apoptosis via loss of mitochondrial membrane potential and activation of MAPK signaling pathway	[88]
	Ophiobolin A	Sesterterpenoid	*Bipolaris orizae*	GL19 cells (glioblastoma), U373-MG cells (glioblastoma)	Induction of paraptosis-like cell death via mitochondrial damage and reduction on BKCa channel activity	[89]
	Pediocin	Bacteriocin	*Pediococcus acidilactici* K2a2-3	HT-29 cells (colorectal cancer), HeLa cells (cervical cancer)	Induction of cell death	[90]
	Plantaricin	Bacteriocin	*Lactobacillus plantarum*	SW480 cells (colorectal cancer)	Induction of apoptosis via caspase-3 activation and PARP-1 cleavage	[91]
	Pradimicin-IRD	Polycyclic Antibiotic	*Amycolatopsis* sp. IRD-009	HCT-116 cells (colorectal cancer)	Cell cycle modulator (G_0_/G_1_ phase arrest), induction of apoptosis via DNA double strand interaction and damage	[92]
	Prodigiosin	Tripyrrole Pigment	*Serratia marcescens*	MDA-MB-231 cells and xenograft (breast cancer), MDA-MB-468 cells (breast cancer)	Induction of apoptosis and downregulation of HSP90α. Suppression of migration and invasion and induction of apoptosis and via inhibition of Wnt/ β-catenin signaling	[93, 94]
	Pyocin S2	Bacteriocin	*Pseudomonas aeruginosa*	HepG2 cells (hepatocellular carcinoma), IM-9 cells (B-lymphoblastoid cell line)	Induction of cell death	[95]
	Pyocyanin	Phenazine Pigment	*Pseudomonas aeruginosa*	MCF7 cells (breast cancer)	Induction of apoptosis and necrosis	[96]
	Rebeccamycin/NSC 655649	Indocarbozole	*Lechevalieria aerocoloigenes, Saccharothrix aerocolonigenes*	P388 cells (leukemia), L1210 cells (leukemia), B16 tumor model (melanoma), A549 xenograft (lung adenocarcinoma),	Intercalates with DNA, promotes double strand breakage, inhibits topoisomerase I	[97, 98]
	Resistomycin	Pentacyclic Polyketide Antibiotic	*Streptomyces aurantiacus* AAA5	HepG2 cells (hepatocellular carcinoma), HeLa cells (cervical cancer), MDA-MB-231 xenograft (breast cancer), patient derived xenograft (breast cancer)	Induction of cell death. Suppression of tumor progression, metastasis, and invasion by Pellino-1 inhibition and SNAIL/SLUG degradation	[99, 100]
	Salinomycin	Carboxylic Polyether Ionophore	*Streptomyces albus*	OVCAR-8 (ovarian cancer), mammary cancer stem cells, patient -derived CLL cells	Induction of apoptosis and G1 cell cycle arrest via Skp2 destabilization and Stat3 inactivation mediating p27Kip1 accumulation. Accumulation of iron in lyososomes to promote ROS generation. Induction of apoptosis via inhibition of Wnt/β-catenin signaling cascade	[101–103]
	Thioholgamide A	Peptide	*Streptomyces* sp. *MUSC 136* *T*	HCT-116 cells (colorectal cancer), RIL175 cells (hepatocellular carcinoma), HUH-7 cells (hepatocellular carcinoma), MCF-7 cells (breast cancer)	Induction of caspase dependent apoptosis via impairment of mitochondrial function and oxidative phosphorylation	[104]
	Tubercidin	Adenosine Analog	*Streptomyces tubercidicus*	DMS 114 cells and xenograft (small cell lung carcinoma)	Induction of apoptosis and downregulation of BCAT1	[105]
	VM48130	Macrolide Antibtiotic	*Streptomyces* sp. FJS31-2	A549/DDP cells (lung adenocarcinoma)	Induction of cell death, downregulation of drug-resistance related genes	[106]
	Violacein	Bis-indole Pigment	*Chromobacterium violaceum*	HT29 cells (colorectal cancer), HL60 cells (acute promyelocytic leukemia)	Induction of cell death, downregulation of EGFR and AXL signaling. Induction of apoptosis via TNFR1/NF-κB signaling	[107, 108]
Wentilactone A Wentilactone B	Wentilactone A, B	Tetranorditerpenoids	*Aspergillus wentii* EN-48	NCI-H460 cells and xenograft (lung large cell carcinoma), NCI-H4466 cells and xenograft (small cell lung carcinoma), SMMC-7721 cells and xenograft (hepatocellular carcinoma)	Cell cycle modulator (G2/M arrest), induction of apoptosis via mitochondrial damage and targeting HRAS-GTP to activate the Ras/Raf/ERK/p53-p21 signaling cascade Induction of apoptosis via ROS generation and Ras-dependent activation of MAPK signaling pathway	[109, 110]

**Table 2:** Clinical trials incorporating microbial metabolites.

Drug Name	Class	Mechanism of Action	Phase	Indication	Route of Administration	Therapeutic Strategy	Results (if completed)	Trial Number	Reference
Annamycin	Anthracycline Antibiotic	Topoisomerase II Inhibitor	I/II	Acute Myeloid Leukemia	Intravenous	Monotherapy	Good safety profile, establishment of RP2D	NCT03388749	[111]
Camidanlumab Tesirine	Antibody-drug Conjugate Containing Pyrrolobenzodiazepine Antibiotic	Targeting CD25 for selective induction of cell death via DNA crosslinking	I	Relapsed/Refractory Lymphoma	Intravenous	Monotherapy	Grade ≥3 treatment-emergent adverse event occurred in 76.7% of patients. Response to therapy occurred in 86.5% of classic Hodgkin’s lymphoma patients at 45 μg/kg dosage	NCT02432235	[112]
EC1169	Tubulysin B Hydrazide conjugated to Prostate-Specific Membrane Antigen-Specific Ligand	Binding to prostate-specific membrane antigen, to induce selective cell death	I	Solid Tumors	Intravenous	± EC0652	N/A	NCT02202447	[113]
Inosine	Purine Nucleoside	Enhancement of antitumor CD8 + T cell function	II	Solid Tumors	Oral	+ PD-1/PD-L1 inhibitor ± Chemotherapy/targeted therapy	N/A	NCT05809336	[45, 47]
LMB-2	Anti-CD25 antibody-drug conjugate containing truncated Pseudomonas exotoxin A	Targeting CD25 for selective induction of cell death	I/II	Adult T Cell Leukemia	Intravenous	+Fludarabine +Cyclophosphamide	Active Trial	NCT00924170	[114]
Marizomib	Proteasome Inhibitor	Inhibition of chymotrypsin-like, trypsin-like, and caspase-like subunits of the proteasome	I/II	Malignant Glioma	Intravenous	Monotherapy (part A) +Bevacizumab (part B)	Good safety profile, however, no improvements of clinical outcomes with addition of marizomib	**NCT02330562**	[115]
Mifamurtide	Mycobacterium muramyl dipeptide derivative	Immunostimulation via activation of pattern recognition receptor signaling	II	Advanced Osteosarcoma	Intravenous	± Ifosfamide	No complete or partial responses from mifamurtide treatment. Increase in macrophage activation factor, G-CSF	NCT02441309	[116]
Moxetumomab Pasudotox	Anti-CD22 antibody-drug conjugate containing Pseudomonas exotoxin A	Targeting CD22 for selective induction of cell death	III	Relapsed/Refractory Hairy Cell Leukemia	Intravenous	Monotherapy	Good safety profile. Complete response to therapy occurred in 41% of patients, improved progression free survival of 41.5 months	NCT01829711	[117]
P28	Redox protein derived from Pseudomonas aeruginosa	Cancer-cell selective peptide that blocks COP1 binding to p53, inducing cell cycle arrest	I	Pediatric Central Nervous System Tumors	Intravenous	Monotherapy	Well tolerated. A patient subset experienced stable disease, however, no complete or partial responses observed	NCT01975116	[118]
Plinabulin	Diketopiperazine Derivative	Tubulin polymermization inhibitor	I	Metastatic Non-Small Cell Lung Cancer	Intravenous	+ Nivolumab	Active Trial	NCT02812667	[119]
Ridaforolimus	Rapamycin Analog	mTOR inhibitor	II	Breast Cancer	Oral	+ Exemestane ± Dalotuzumab	Grade ≥3 adverse events occurred 67.5% of patients in R/E arm, and 59% in R/D/E arm. R/D/E did not improve clinical outcomes compared to R/E	NCT01605396	[120]
SYD1875	Antibody-drug conjugate containing DNA alkylating molecule duocarmycin	Targeting 5T4 for selective induction of cell death via DNA alkylation	I	Solid Tumors	Intravenous	Monotherapy	N/A	NCT04202705	[121]
Tanespimycin	Geldanamycin Derivative	HSP90 inhibition to induce apoptosis	II	Thyroid Cancer	Intravenous	Monotherapy	N/A	NCT00118248	[122]
Temsirolimus	Rapamycin Derivative	mTOR inhibitor	I/II	Relapsed/Refractory Lymphoma	Intravenous	+ Lenalidomide	Grade ≥3 adverse events were common. Overall response to therapy occurred in 64.1% of patients.	NCT01076543	[123]
UCN-01	Staurosporine Analog	Protein kinase C inhibitor, cell cycle modulator (G_1_ phase arrest)	II	T cell Lymphoma	Intravenous	Monotherapy	Adverse events occurred in 95% of patients between cohorts. Complete and partial responses to therapy occurred 18% and 9% of patient cohort	NCT00082017	[124]
Valrubicin	Anthracycline Analog	Topoisomerase II Inhibitor	III	Bladder Cancer	Intravesical	Monotherapy	N/A	NCT01310803	[125]

### Azurin-p28

Azurin-p28 is a redox copper protein produced by gram-negative bacteria such as *Alcaligenes dentrificans* and *Pseudomonas aeruginosa*. Initial studies using azurin and its derivative peptides as a potent inducer of cell death, while maintaining selective cytotoxicity to cancer cells. Two mechanisms for the selectivity of azurin have been described. First, azurin has been shown to interfere with EPH receptor B2 signaling, a receptor upregulated in prostate cancer, gastrointestinal malignancies, and glioblastoma [35]. In addition, the p28 domain of azurin preferentially penetrated melanoma UISO-Mel-2 cells over noncancerous fibroblasts through caveolin-mediated internalization, suggesting p28 targets a receptor is highly expressed on cancer cells [36]. Azurin-mediated cytotoxicity occurred through the formation of an azurin and p53 complex, resulting in stabilization of p53, ultimately increasing cytosolic Bax and cytochrome c levels to initiate apoptosis [37]. The stabilization of p53 by azurin was later discovered by Hu et. al to be a result of overlap between azurin binding sites on p53 and that of E3 ubiquitin ligase COP1. Consequently, the competitive binding of azurin to p53 prevents COP1 ubiquitination and proteasomal degradation [38].

### Butyrate

Butyrate is a short-chain fatty acid commonly produced by human gut microbiota such as *Faecalibacterium prausnitzii* and *Clostridium leptum*, through bacterial fermentation of indigestible dietary fiber. The anticancer effect of butyrate is dynamic, in that it demonstrates different effects to different cell types. Direct treatment of colorectal cancer cells with butyrate has been shown to trigger apoptosis through activation of the JNK/AP-1 pathway [39]. In a separate study, butyrate treatment inhibited colony formation and cell migration while also inducing apoptosis through downregulation of homeostasis regulatory gene, thioredoxin-1 [40]. Though inducing cell death in cancer cells, butyrate enhances the tumoricidal properties of T cells. Through metabolomic screening of gut microbial contents for potential anticancer mediators, butyrate was identified on the basis it most strongly promoted effector T cell responses of all metabolites screened. Butyrate promoted granzyme B and IFN-γ production from CD8 + T cells while at the transcriptional level downregulated exhaustion-associated genes, suggesting butyrate may also be able to reinvigorate T cells in addition to promoting effector functionality. Most strikingly, however, was that in vivo butyrate treatment enhanced the antitumor effects of immunogenic cancer therapies such as oxaliplatin and anti-PD-1 blockade, through its CD8 + T cell boosting effect [41].

#### Cordycepin

Cordycepin, or 3’deoxyadenosine, is a bioactive isolate from the fungus Cordyceps militaris. Structurally, cordycepin resembles the nucleoside adenosine, but the key difference lies in the substitution of a hydrogen atom for the hydroxyl group at the 3’ position. Due to its structural resemblance to adenosine, cordycepin exhibits the ability to disrupt cell proliferation and viability across various types of solid tumors. Cordycepin has consistently demonstrated anticancer effects across various cancer types by modulating the Akt pathway signaling, indicating a reproducible mechanism for its therapeutic impact. The induction of anticancer effects by cordycepin has been consistently observed in diverse cancer types, with evidence pointing towards the modulation of Akt pathway signaling as a consistent and underlying mechanism [42]. In ovarian cancer cells, cordycepin induced apoptosis in a dose-dependent manner due to reduced CCL5 release to subsequently attenuate Akt/NF-kB signaling. Similarly, esophageal cells treated with cordycepin experienced apoptosis, in addition to reduced migratory and invasive capacity via AMPK activation and inhibition of Akt [43]. To build upon the intrinsic anticancer effects of cordycepin, a cordycepin-based ProTide has been developed. NUC-7738 is a ProTide that comprises cordycepin with a phosphoramidate transformation to augment potency and stability of the compound. These enhancements have positioned NUC-7738 to be used in a first-in-human phase I clinical trial in patients with advanced solid tumors including melanoma, colorectal, gastric, and lung malignancies. Dose-escalation of NUC-7738 demonstrated a favorable safety profile with low-grade treatment-related adverse effects, as well as stabilizing disease progression in immunotherapy-resistant tumor types [44].

### Inosine

Inosine is a nucleoside metabolite that consists of hypoxanthine linked to a ribose ring, produced during adenosine deamination. Though ubiquitous with purine metabolism, inosine produced by the gut residing bacterium *Bifidobacterium pseudolongum* has demonstrated a role as a potential antitumor immunotherapy [45]. In germ free mice bearing MC38 tumors (murine colorectal carcinoma cell line), the administration of inosine, CpG as a costimulator, and anti-CTLA-4 therapy experienced a significant reduction in tumor burden, demonstrating the ability of microbiome-derived metabolites to potentiate the response to immune checkpoint blockade therapy [46]. The antitumor effect of inosine was found to be largely T-cell intrinsic, increasing populations of intratumoral IFN-γ + T cells and promoting Th1 differentiation through A_2a_R signaling [45]. Paralleling its canonical role in purine metabolism, inosine also has been demonstrated as a carbon source for antitumor T cells. Activated CD8 + T cells in the presence of inosine alone experienced enhanced tumoricidal activity and cell proliferation mediated through pyruvate nucleoside phosphorylase. The high demand for glucose by cancer cells in the tumor microenvironment restricts its availability for effector cells. Consequently, inosine serves as an alternative fuel source, enabling T cells to exert their cytotoxic functions within the tumor [47].

### Propionate

Propionate is a short chain fatty acid produced from several human gut microbiota including *Veillonella parvula, Bacteroides eggerthii, Bacteroides fragilis, Ruminococcus bromii*, and *Eubacterium dolichum*. Though sharing many characteristics with butyrate, propionate appears to have more of an ambiguous role in cancer. When administered orally, and thus interacting with the gut, propionate significantly reduced tumor development in a murine breast cancer xenograft model, through inhibition of JAK2-STAT3 signaling and ROS generation [48]. CD8 + T cells cultured directly with propionate experienced a substantial increase in the release of effector molecules while also reducing HDAC activity, suggesting that potential anticancer effects of propionate may emerge through modulation of T cell responses [49]. In contrast, high systemic propionate levels have also been linked to resistance to therapy in cancer patients. High serum propionate levels was inversely correlated to progression free survival and overall survival as well as associated with poor response to therapy in cancer patients receiving the immune checkpoint inhibitor, ipilimumab [50]. Further, propionate metabolism has been shown to allow breast cancer cells to undergo pro-metastatic programming through accumulation of metabolic byproducts propionyl-CoA and methylmalonic acid. The overexpression of propionyl-CoA dramatically increased the invasive and migratory properties of MDA-MB-231 cells, as well as increasing lung metastasis in a murine tumor model [51]. These contrasting reports of propionate in cancer suggests that route of administration and localization of propionate plays a significant role in determining its pro- or anti- tumor activity.

### Nisin

Nisin is a small, 34 amino acid bacteriocin that is produced through fermentation by Gram-positive bacterium *Lactococcus lactis*. Due to its broad-spectrum antibacterial properties, nisin has canonically been used as a food preservative, however, as nisin is also a bacteriocin, its anticancer potential has become an area of interest. Initial cytotoxicity studies describe nisin to preferentially arrest cell proliferation and induce apoptosis in HNSCC cells over noncancerous oral keratinocytes- which were unaffected by nisin treatment. The pro-apoptotic and antiproliferative effects of nisin were found to be mediated through activation of pro-apoptotic cation transport regulator CHAC1 [52]. The tumor-selective qualities of nisin were further confirmed using an oral cancer xenograft mouse model. Nisin-treated animals experienced a dose-dependent reduction in tumor volume and intratumoral microvessel density, while exhibiting no histological signs of toxicity to the liver, lung, or kidney [53]. The role of nisin as an adjuvant therapy to established anticancer agents has also been investigated. Paralleling these findings, the combination of nisin and doxorubicin drastically increased the rate of apoptosis in cancerous tissue [54]. In all, nisin holds much promise as a minimally toxic cancer therapy that can be used as both a standalone therapy and in combination.

### Microbial metabolites as immunomodulatory agents

A fundamental function of the immune system is the ability to distinguish “self” from “non-self.” Upon recognition of foreign or “non-self” antigens, the immune system incites a robust, inflammatory response in attempt to eradicate the foreign particles. Given the microbial sourcing, thus “non-self” origin of these compounds, the immunostimulatory abilities of these metabolites are being investigated to utilize the power of the immune system to fight cancer. Recently, microbial metabolites produced from both the gut and tumor microbiome, have been recently identified for their robust immunomodulatory potential.

**Fig. 3:**
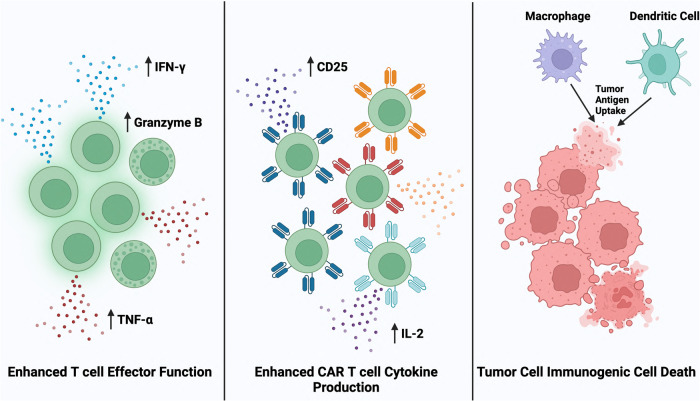
Mechanisms of anticancer immunomodulation by microbial metabolites. Direct interaction between microbial metabolites pentanoate and trimethylamine N-oxide (TMAO) and CD8 + T cells increases the release of effector T cell associated cytokines such as IFN- γ and TNF-alpha. In addition, these microbial metabolites promote the production of cytolytic granules in cytotoxic T cells. In CAR T cells, microbial metabolite pentanoate enhances the production of CD25 and IL-2, a cytokine most commonly released by activated CD4+ and CD8 + T cells. Tumor cell death via chromomycin A5 can lead to the release of tumor-associated antigens as well immunostimulatory molecules such as damage associated molecular patterns. These components of tumor cells can be taken up by macrophages and dendritic where the antigen is processed to be presented to T cells, inducing tumor-specific immunity. Created with Biorender.com.

Pentanoate, a short chain fatty acid produced by gut residing bacterium *Megasphaera massiliensis*, drastically increased the frequency of effector IFN-γ+ and TNF-α + CD8 + T cells in vitro. Using the B16 melanoma model, tumor bearing mice underwent adoptive transfer of pentanoate cultured CD8 + T cells, leading to a dramatic reduction in tumor growth. Paralleling that result, tumors of mice treated with pentanoate cultured T cells had significant infiltration of additional effector IFN-γ+ and TNF-α + CD8 + T cells, as well as increased expression of effector marker CD25 [49]. The treatment of T cells with pentanoate may have potential for clinical application, as human CAR T cells treated with pentanoate also enhanced CD25 expression as well as IL-2 production [49] (Fig. 3).

Enhanced antitumor immune cell function has also been demonstrated by trimethylamine N-oxide (TMAO), a choline metabolite produced by *Blautia, Ruminococcus*, *Faecalibacterium, Dorea, Tyzzerella*, and *Roseburia* species. Wang et. al discovered that TMAO produced by tumor-residing species was associated with an immune-activated tumor microenvironment in patients with triple negative breast cancer [55]. Similarly, breast tumor bearing mice treated with TMAO experienced a drastic reduction in tumor growth [55]. TMAO in combination with anti-PD-1 therapy also significantly enhanced the efficacy of immune checkpoint blockade, suggesting T cell activity may contribute to the antitumor response of TMAO. This was supported as TMAO-treated tumors had a significant increase in CD8 + T cell infiltration. In addition, CD8 + T cells from TMAO-treated tumors had enhanced production of effector cytokines IFN-γ and TNF-α [55].

## Microbial-based bioactive compounds

Secondary metabolites from microbes encompass only a fraction of biologically active natural products that have the potential to treat human disease. In its simplest form, biologically active compounds are compounds that have a physiological effect an organism. Thus, these compounds have been investigated for therapeutic potential. Tryptophan derivative indole-3-lactic acid (ILA) produced by *Lactobacillus gallinarum* and *Lactobacillus plantarum* is a bioactive tryptophan derivative with promising anticancer properties. Oral administration of ILA to Apc^Min/+^ C57BL/6 mice drastically prevented spontaneous colon tumor formation as well as reducing tumor size of established tumors. This effect was a result of apoptosis mediated by ILA in colorectal cancer cells, but not in non-cancerous epithelial cells [56]. In another study, ILA was shown to have immune-mediated antitumor properties to target colorectal cancer. ILA enhanced CD8 + T cell priming through increased IL-12 production from dendritic cells. Acting directly on tumor infiltrating CD8 + T cells, ILA also increased granzyme B and IFN-γ production [57].

Microbe-produced exopolysaccharides (EPS) are long-chain, high molecular weight polysaccharides currently being investigated for anticancer and immunomodulatory potential [58]. Dietary consumption of EPS derived from *Lactobacillus delbrueckii*, EPS-R1, sensitized Colon26 and 4T1 tumors to anti-CTLA4 blockade therapy, significantly reducing tumor progression [59]. This response occurred in CCL20+ tumors, and accordingly enhanced migration of IFN-γ producing CXCR3 + CCR6 + CD8 + T cells into the tumor. Further, the antitumor CCR6 + CD8 + T cell population was generated through EPS-R1 and the lysophosphatidic acid receptor 2 expressed on CD8 + T cells [59].

## Conclusions and future perspectives

Balancing the effectiveness of cancer treatments with potential side effects presents a significant challenge in cancer therapeutics. Some well-established microbial metabolites, such as doxorubicin and bleomycin, have demonstrated strong abilities to kill cancer cells and are routinely used in cancer treatment plans. These well-established, microbial-derived compounds are often accompanied by severe adverse toxicities. Due to these toxicities, treatment with the compound is often shortened or stopped, or the dosage is lowered to where it becomes significantly less effective in reducing the patient’s disease progression. A solution to these shortcomings with the current microbial-based treatment modalities has been a refocusing towards microbial-based anticancer agents with a high degree of cancer specificity.

Given that these compounds originate from microorganisms there may be good reason for utilizing them for cancer treatment by modulating the immune system. The innate immune system can be engaged by metabolites through pattern recognition receptors that act as sensors, for shared patterns found in microbes. When these receptors are activated, they can trigger a response leading to the activation of antigen-presenting cells and the production of inflammatory and effector cytokines. In the context of cancer, this response can alert the system to the presence of a tumor. Potentially contribute to its elimination. These compounds exhibit a remarkable feature - they contain components that can stimulate the immune system. This immunostimulatory property enables them to precisely target cancerous tissues, leaving healthy tissues unaffected.

Similarly, the selectivity of the immune system can also be utilized in the context of antibody-drug conjugates incorporating microbial metabolites as a “warhead” molecule. In this context, a highly and non-selectively cytotoxic microbial metabolite would be conjugated to a monoclonal antibody. To selectively target cancer cells, the antibody is specific for moieties expressed only on malignant cells. As a result, the cytotoxic compound is solely delivered to cancerous cells, leading to highly selective cancer cell death. These compounds have already demonstrated clinical efficacy and will likely be further developed through incorporation of novel tumor antigen targets and microbial metabolites with greater potency.

This exciting generation of secondary metabolites represents just a fraction of the bioactive compounds found in the microbial world that hold potential for treating human diseases, including cancer.
